# Patients with migraine are right about their perception of temperature as a trigger: time series analysis of headache diary data

**DOI:** 10.1186/s10194-015-0533-5

**Published:** 2015-05-26

**Authors:** Albert C. Yang, Jong-Ling Fuh, Norden E. Huang, Ben-Chang Shia, Shuu-Jiun Wang

**Affiliations:** Department of Psychiatry, Taipei Veterans General Hospital, Taipei, Taiwan; Division of Psychiatry, School of Medicine, National Yang-Ming University, Taipei, Taiwan; Center for Dynamical Biomarkers and Translational Medicine, National Central University, Chungli, Taiwan; Division of Interdisciplinary Medicine and Biotechnology, Beth Israel Deaconess Medical Center/Harvard Medical School, Boston, MA USA; Department of Neurology, Neurological Institute, Taipei Veterans General Hospital, No. 201, Sec. 2, Shih-Pai Road, Taipei, 11217 Taiwan; Division of Neurology, School of Medicine, National Yang-Ming University, Taipei, Taiwan; Preparatory office in Big Data Research Center & School of Management, Taipei Medical University, Taipei, Taiwan

**Keywords:** Empirical mode decomposition, Migraine, Time-dependent intrinsic correlation, Weather, Temperature

## Abstract

**Background:**

Researches to date on the association between headache and weather have yielded inconsistent results. Only a limited number of studies have examined the clinical significance of self-reported weather sensitivity. This study aimed to identify the difference in the association of headache with temperature between migraine patients with and without temperature sensitivity.

**Methods:**

66 migraine patients (75.8 % female; mean age 43.3 ± 12.9 years) provided their 1-year headache diaries from 2007 to a headache clinic in Taipei, Taiwan. 34 patients (51.5 %) reported sensitivity to temperature change but 32 (48.5 %) did not. Time series of daily headache incidence was modeled and stratified by temperature sensitivity. Empirical mode decomposition was used to identify temporal weather patterns that were correlated to headache incidence, and regression analysis was used to examine the amount of variance in headache incidence that could be explained by temperature in different seasons.

**Results:**

Among all migraine patients, temperature change accounted for 16.5 % of variance in headache incidence in winter and 9.6 % in summer. In winter, the explained variance increased to 29.2 % among patients with temperature sensitivity, but was not significant among those without temperature sensitivity. Overall, temperature change explained 27.0 % of the variance of the mild headache incidence but only 4.8 % of the incidence of moderate to severe headache during winter.

**Conclusions:**

This diary-based study provides evidence to link the perception of temperature sensitivity and headache incidence in migraine patients. Those who reported temperature sensitivity are more likely to have headache increase during the winter, particular for mild headaches.

## Background

The perception that weather can trigger headaches is widely reported in patients with migraine or tension-type headache [1–5]. Researchers have provided considerable yet mixed evidence in this regard [6]. Some reports suggest that several inconsistent meteorological factors may be associated with headache, including ambient temperature [5, 7–10], barometric pressure [5, 7, 8, 11, 12], relative humidity [5, 9], and wind speed [13, 14], while other studies show no connection between headache and weather [15–18].

The problems of identifying weather as a trigger in headache patients have been reviewed recently [19, 20]. Studies of the association between headache and weather factors are challenged by the coexistence of multiple headache triggers, varied time lags between triggers and headache onset, and heterogeneous headache populations [19, 20]. Weather factors may interact with each other and operate synergistically to trigger headache [5]. Moreover, measurements of weather variables often show complex fluctuations over time, thus their associations with headache are difficult to analyze with conventional methods due to presence of multiple intrinsic components and collinearity among weather variables.

Recently, we combined an adaptive-based method of empirical mode decomposition (EMD) [21, 22] and regression methods to delineate temporal relationships between headache incidence and weather changes utilizing data from a 5-months headache diary [23]. The EMD method decomposes weather data into a set of intrinsic oscillations, called intrinsic mode functions (IMFs), that are orthogonal to each other and each represents a certain mode of weather fluctuations. Using EMD analysis, we found a temporal association of increased headache incidence with temperature IMFs that coincided with cold fronts during the winter [23].

In this study, we further sought to test the myth of the perception of temperature sensitivity in migraine patients, based on a cohort of migraine patients with or without perception of temperature sensitivity and the EMD analysis of the association between headache incidence and temperature using 1-year headache diary data. We hypothesized that headaches reported from migraine patients perceiving a temperature trigger were associated with temperature changes, and such association may be reduced in those who do not perceive a temperature trigger.

## Methods

### Patients

A total of 66 patients with migraine were included in this study (50 females, 75.8 %; mean age 43.3 ± 12.9 years). The diagnosis of migraine (coded 1.1, 1.2, 1.5.1, 1.6) was based on the International Classification of Headache Disorders, 2^nd^ edition, 2004 (ICHD-2) [24]. Patients who lived within the Taipei metropolitan area were recruited from the headache clinic in the Department of Neurology, Taipei Veterans General Hospital. The headache clinic has operated since 1997. All patients completed a headache intake form and received headache diagnoses by headache specialists. They were asked to keep a headache diary for diagnostic and treatment purposes. The headache diary is a routine practice in headache clinics and is normally not for research purposes; hence the patients recruited in the present analysis were blind to the study protocol. We copied the patients’ diaries as part of the history records during their return visits.

Patients had their headache diary for a minimum of 7 months during the period from January 1^st^, 2007 to December 31^st^, 2007 were recruited for these analyses. The patients were asked to record their headache diary on a daily basis, which was used to calculated the daily incidence of headache as the proportion of patients reporting headache out of the total number of patients on each day. The intensity of headache was recorded as mild, moderate or severe in the headache diary. Of note, at their first visit to this headache clinic patients were also asked in a headache intake form whether they felt that their headaches were vulnerable (or worsened) due to temperature changes based on their personal perception. Thus, we correlated the time series of daily headache incidence with the temperature IMF through the whole year with and without the self-reported temperature sensitivity.

### Weather data

The Central Weather Bureau, Taiwan, provided daily temperature data in the central Taipei area. Table 1 summarizes the weather data in year 2007 in Taipei City. Taipei City locates in sub-tropical regions and has a marine climate which characterized by a cool winter and a hot summer; note that the weather changes among seasons are dominant by temperature fluctuations. The annual averaged temperature in Taipei City is about 23 °C, ranging from 18 °C in winter to 29 °C in summer. Therefore, we chose temperature as the target meteorological variable to be associated with headache incidence data.

**Table 1:** Summary of weather variables in year 2007 at Taipei City, Taiwan

Variables	Whole year	Spring (Mar-May)	Summer (Jun-Aug)	Autumn (Sep-Nov)	Winter (Dec-Feb)
Temperature, °C	23.6 ± 5.0	22.5 ± 4.4	29.1 ± 1.8	24.4 ± 3.3	18.5 ± 2.5
Pressure, mmHg	1012.2 ± 6.9	1012.7 ± 4.5	1005.2 ± 3.6	1012.1 ± 7.0	1019.2 ± 3.7
Humidity, %	75.6 ± 8.9	74.8 ± 8.8	74.8 ± 8.3	77.6 ± 8.5	75.1 ± 9.7
Sunshine duration, hour	3.9 ± 3.7	3.6 ± 3.9	4.9 ± 3.2	3.4 ± 3.7	3.5 ± 3.7
Wind speed, m/s	2.4 ± 1.3	2.1 ± 1.1	1.7 ± 1.1	3.3 ± 1.3	2.6 ± 1.1

### Empirical Mode Decomposition (EMD)

The EMD was developed to decompose and identify the intrinsic oscillations, termed IMF, that are embedded in a time series [21]. The decomposition was carried out by a sifting process to decompose original time series into a finite set of IMFs. The detail of the EMD method [21] and its application to epidemiological time series was previously described [23, 25–27]. In brief, the sifting process is comprised of the following steps: 1) connecting the local maxima or minima of a targeted signal to form the upper and lower envelopes by natural cubic spline lines; 2) extracting the first prototype IMF by estimating the difference between the targeted signal and the mean of the upper and lower envelopes; and 3) repeating these procedures to produce a set of IMFs that are represented by a certain frequency-amplitude modulation at a characteristic time scale. The decomposition process is complete when no more IMFs can be extracted, and the residual component is treated as the overall trend of the raw data.

The advantage of EMD method is to remove noise and non-stationary oscillations (for example, secular trends) that are irrelevant to the temporal weather events. Furthermore, IMF has a zero-mean distribution, thereby reducing type I statistical error in the subsequent regression analysis. This study used a publicly available EMD algorithm based on Matlab software (version 2007; The Mathworks, Natick, Massachusetts) (http://rcada.ncu.edu.tw/research1.htm).

### Statistical analysis

SPSS for Windows Version 15.0 (Chicago, IL; SPSS Inc.) software was used for statistical analyses. One-way analysis Of variance (ANOVA) was used to test for differences in headache incidence among seasons and post hoc least significant difference (LSD) tests were used for paired-group comparisons.

**Fig. 1:**
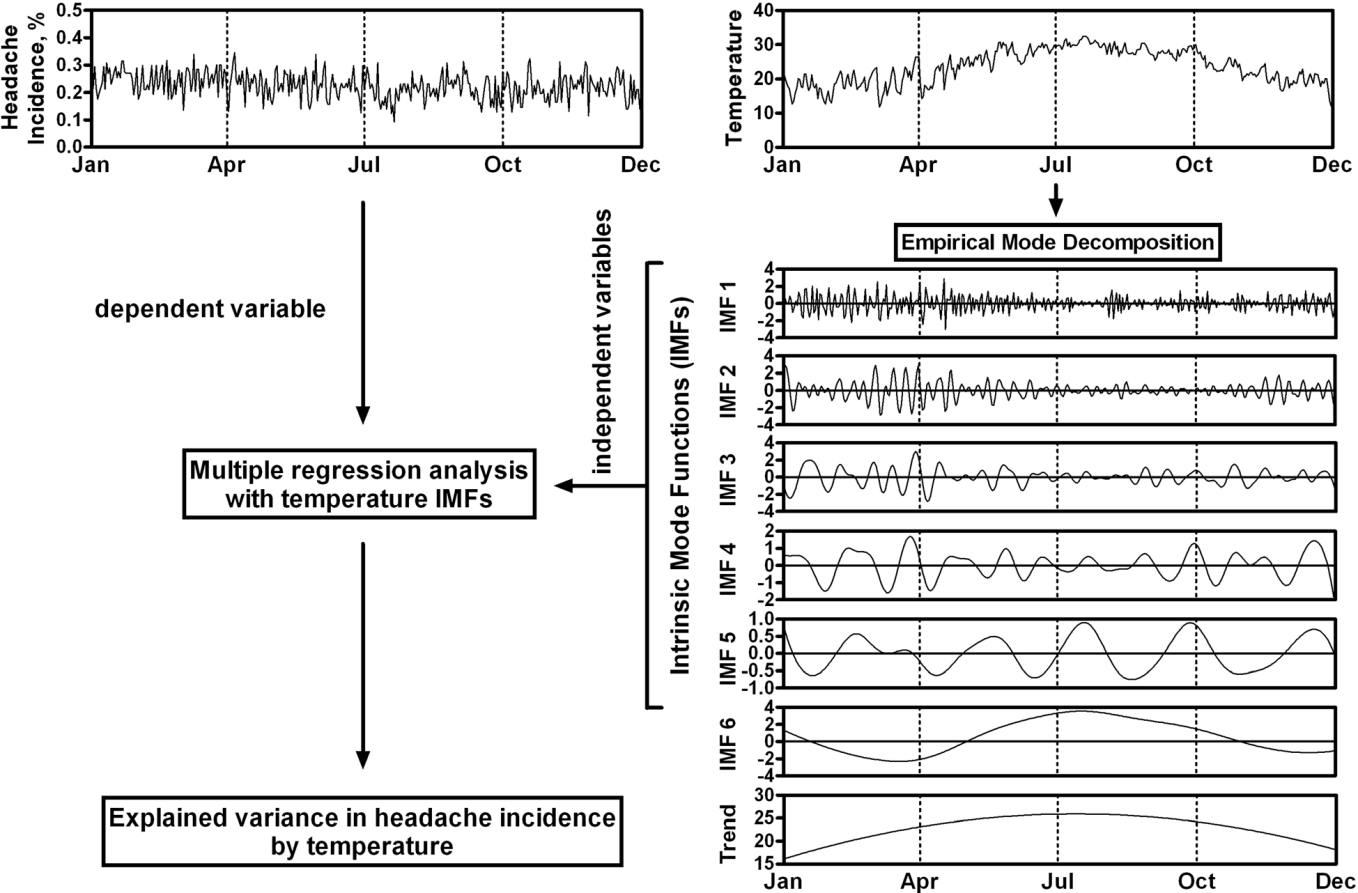
Flow chart of time series analysis of headache and temperature data. Using empirical mode decomposition method, temperature time series was decomposed into an overall trend and various modes of fluctuations, termed intrinsic mode functions (IMFs). Each IMF represents a specific mode of temperature fluctuations. These IMFs were correlated with headache incidence data by multiple regression analysis to derive the explained variance in headache incidence by temperature

Figure 1 illustrated the flow chart of time series analysis conducted in this study. The analysis procedures were conducted separately in time series models stratified by status of temperature sensitivity (i.e. temperature sensitizer vs. temperature non-sensitizer groups). The analyses comprised two parts. The first part of the analysis involved the use of EMD to decompose temperature data into IMFs, thus separating temperature time series data into various modes of fluctuations operating at different time scales, as shown in Fig. 1. The second part of analysis is to delineate the association of daily headache incidence with certain weather modes, that is, the key temperature components that are correlated to daily headache incidence.

Linear regression analysis with a forward stepwise method was used to study the association between daily headache incidence (dependent variable) and decomposed temperature IMFs (independent variable). In each temperature regression model, the variance inflation factor (VIF) was estimated for all temperature IMFs and a VIF value of 10 or greater is considered to be an indication of significant collinearity, and the R square was determined to estimate the variance in daily headache incidence that could be explained by the identified IMFs. Moreover, we analyzed the data from the entire study period, as well as from each season, including spring (March to May), summer (June to August), autumn (September to November), and winter (December to February). A 2-tailed p-value of less than 0.05 was required for statistical significance in the regression analyses.

## Results

### Patients

Table 2 summarizes the demographic and clinical characteristics of study subjects. Of the 66 participants, 63 (95.5 %) were diagnosed as having migraines, and 3 (4.5 %) probable migraine. Mean age of onset was 24.5 ± 13.8 years with duration of illness 18.8 ± 13.6 years. For acute treatment, 42 patients (63.6 %) were on sumatriptan and 37 (56.1 %) on non-steroidal anti-inflammatory drugs. Regarding preventive agents, 40 (60.6 %) were on propranolol, 20 (30.3 %) on anticonvulsants (topiramate or valproic acid), 13 (19.7 %) on flunarizine, 12 (18.1 %) on antidepressants (venlafaxine, duleoxetine, mirtazapine, or amitriptyline). As for other known medical conditions, 6 patients (9.1 %) had diabetes, and 2 (3.0 %) hypertension.

**Table 2:** Demographic and clinical characteristics

Characteristics	Total	Temperature sensitive	Temperature non-sensitive	P*
	N = 66	N = 34	N = 32	
Age, year	43.3 ± 12.9	42.6 ± 12.3	44.0 ± 13.8	0.675
Female, N (%)	50 (75.8 %)	27 (79.4)	23 (71.9)	0.671
Age of onset, year	24.5 ± 13.8	20.3 ± 9.8	29.1 ± 16.0	*0.009*
Duration of illness, year	18.8 ± 13.6	22.4 ± 13.1	14.9 ± 13.2	*0.025*
Headache days per month, all severity	6.3 ± 6.2	5.4 ± 5.0	7.2 ± 7.4	0.262
Headache days per month, moderate or severe	3.0 ± 3.2	2.1 ± 1.1	4.0 ± 4.2	*0.012*
Acute treatment, N (%)				
Sumatriptan	42 (63.6)	22 (64.7)	20 (62.5)	1.000
NSAIDs	37 (56.1)	22 (64.7)	15 (46.9)	0.225
Preventive treatment, N (%)				
Propranolol	40 (60.6)	20 (58.8)	20 (62.5)	1.000
Anti-convulsants	20 (30.3)	12 (35.4)	8 (25.0)	0.522
Flunarizine	13 (19.7)	8 (23.5)	5 (15.6)	0.617
Anti-depressants	12 (18.1)	7 (20.6)	5 (15.6)	0.841
Either one	56 (84.8)	27 (79.4)	29 (90.6)	0.198
Hypertension	2 (3.0)	1 (2.9)	1 (3.1)	0.498
Diabetes	6 (9.1)	3 (8.8)	3 (9.4)	1.000

NSAIDs: Non-steroid anti-inflammatory drugs

*Comparisons between temperature sensitive and non-sensitive patients

Thirty-four patients (51.5 %) reported that their headaches were sensitive to temperature change and 32 patients did not. Temperature sensitive patients had significantly earlier age of onset (20.3 ± 9.8 vs. 29.1 ± 16.0; p = 0.009), longer duration of illness (22.4 ± 13.1 vs. 14.9 ± 13.2 years; p = 0.025), and fewer moderate to severe headache days per month (2.1 ± 1.1 vs. 4.0 ± 4.2; p = 0.012) than non-sensitive patients. The two temperature groups did not differ in the other demographic data or headache profiles.

### Headache incidence

In total, 22,298 diary entries documenting 5,009 headache days were recorded during year 2007, including 2,640 mild, 1,944 moderate, and 425 severe headache days. Forty-two (63.6 %) patients completed the entire year of their headache diary. The mean length of the combined headache diaries was 338 ± 42 days (range: 229–365 days). The mean headache days per month were 6.3 days (all levels of intensity). Mean daily headache incidence was 22.5 ± 4.7 % (range: 9.2–34.4 %).

Table 3 summarizes the influence of seasons on headache incidence between temperature groups. Both temperature groups show significant between-seasons differences in headache incidence. Post-hoc comparisons indicate that temperature sensitive patients had a significantly higher frequency of headache in winter than in the other seasons, while temperature non-sensitive patients had a significantly higher frequency of headache in spring (all p < 0.05).

**Table 3:** Mean daily headache incidence (%) in each season in year 2007

Groups	Whole year	Spring^1^ (Mar-May)	Summer^2^ (Jun-Aug)	Autumn^3^ (Sep-Nov)	Winter^4^ (Dec-Feb)	P*	Post-hoc*
All patients (N = 66)	22.5 ± 4.8	23.8 ± 4.9	21.3 ± 4.3	21.4 ± 4.8	23.6 ± 4.4	<0.001	1 > 2; 1 > 3; 4 > 2; 4 > 3
Temperature sensitive patients (N = 34)	19.7 ± 6.6	19.3 ± 6.4	18.9 ± 5.9	19.1 ± 6.9	21.5 ± 7.1	0.031	4 > 3; 4 > 2; 4 > 1
Temperature non-sensitive patients (N = 32)	25.5 ± 6.5	28.5 ± 6.2	23.7 ± 5.8	23.8 ± 5.9	25.9 ± 6.9	<0.001	1 > 2; 1 > 3; 1 > 4

*Headache incidence of four seasons was compared by ANOVA and LSD post-hoc test

### Identifying the association of headache incidence with temperature between seasons

Regression analysis was conducted separately in four seasons to examine the association of headache incidence with temperature IMFs, as shown in Table 4. For the headache data comprised of all patients, temperature explained 16.5 % of the headache incidence during winter but 9.6 % during summer. However, spring and autumn were not significant. Next, we stratified the headache time series by personal perceptions of temperature sensitivity. For emperature sensitive patients, temperature IMFs accounted for 29.2 % of the variance in headache incidence (R^2^ = 0.292) during winter and 6.0 % during spring (R^2^ = 0.060) but was not significant in summer or autumn. In contrast, for temperature non-sensitive patients, temperature accounted for 14.8 % the variance in headache incidence (R^2^ = 0.148) exclusively during summer.

**Table 4:** Models of headache incidence and temperature IMFs

Group	Model summary (R Square)			
	Spring	Summer	Autumn	Winter
All Patients (N = 66)	NS	0.096 (*P* = 0.003)	NS	0.165 (*P* < 0.001)
Temperature Sensitive Patients (N = 34)	0.060 (*P* = 0.018)	NS	NS	0.292 (*P* < 0.001)
Temperature Non-Sensitive Patients (N = 32)	NS	0.148 (*P* < 0.001)	NS	NS

NS: non-significant *(P* > .0.05)

Furthermore, we examined the relationship between headache intensity and temperature sensitivity. Regression analysis was conducted separately in two groups of headache incidence data stratified exclusively by mild only as well as moderate or severe headache intensity. For temperature sensitive patients, regression results showed that temperature accounted for 27.0 % (p < 0.001) of the incidence of mild headaches and only for 4.8 % (p = 0.038) of the incidence of moderate to severe headaches during winter, but had no association with any intensity of headaches during spring. For temperature non-sensitive patients, regression results showed that temperature accounted for 7.4 % (p = 0.009) of the mild headaches and 4.6 % (p = 0.039) of the moderate to severe headaches during summer.

Regarding the direction of association between headache and temperature, the regression models showed that headache incidence had the inverse correlations with temperature IMFs, either for temperature sensitive patients during winter (IMF 5: r = −0.523, p < 0.001) and spring (IMF 6: r = −0.310, p = 0.003), and for temperature non-sensitive patients during summer (IMF 5: r = −0.384, p < 0.001).

## Discussion

The study showed that headaches in temperature sensitive migraine patients were associated with temperature mainly during cold period (or winter) (R^2^ = 0.292), while temperature non-sensitive patients did not have such association during the same period. In contrast, a weaker association was demonstrated during only hot period (or summer) in temperature non-sensitive patients (R^2^ = 0.148). To our knowledge, this is the first study to show the different profiles of association between temperature and headache in relation to perceived temperature sensitivity among migraine patients.

Previous studies have shown that around 35–50 % of patients with migraine or tension-type headache perceived weather as a trigger of headaches [1–5]. However, studies attempted to identify headache and weather associations often failed to show the difference between weather sensitizers and non-sensitizers [5, 18]. Furthermore, the amount of weather variable that can explain the variance of headache events also varied. For example, in the study of the effect of Chinook wind on headaches showed only relatively low percentage of patients were sensitive to Chinook wind, despite a finding that a majority of study subjects perceived such sensitivity [13].

In addition, our study also showed that temperature was mainly associated with mild headaches but only to a lesser degree for moderate to severe headache. While the headache intensity reported in this study may be masked due to medication treatment, these findings may still provide a hint that may explain why some studies have difficulty in assessing the relationship between weather and headache [19]. The current ICHD-2 diagnosis of migraine attacks specifies the pain intensity to be moderate or severe. However, mild headaches may progress to moderate or severe migraine attacks, and there is evidence showing that mild intensity headaches in migraineurs are of the spectrum of migraine. [28, 29]. Therefore, studies using strict criteria for migraine attacks may overlook or underestimate the association between headache and weather in migraine patients.

The present study found that while headache incidence was inversely associated with temperature, headache incidence was strongly associated with cold period in temperature sensitive migraineurs, and was associated with hot period only in temperature non-sensitive migraineurs at a lesser degree. Such discrepancy in the perception of temperature sensitivity and its association with seasons might be explained by various headache incidences and the perception of temperature fluctuations among different seasons. First, headache incidence was found to be higher in winter than in other seasons among temperature sensitive patients (Table 3). Second, outdoor thermal perception and levels of discomfort due to weather are usually more sensitive in cold season than in hot season [30]. During winter, temperature sensitive patients may easily sense the changes in temperature. However, during summer time, the variation in temperature might be too small to be perceived as a trigger, and the weaker association of headache with temperature in temperature non-sensitive migraineurs may be too little to evoke the perception of temperature as a trigger. Taken together, cold temperature during winter may contribute more to subjective perception of temperature change as a trigger in certain migraine patients.

Of note, the findings of winter sensitization of headache are consistent with our prior community-based study [23] that showed increased headache incidence was associated with temperature changes during winter, which is temporally correlated to the aftermath of documented cold fronts. While the mechanism of the association between temperature and headache remains largely unknown, headache is known to be associated with hemodynamic changes, which might be aggravated by the cold weather. The decrease in temperature during cold fronts either plays a role in precipitating headache attacks or have a priming effect on headache occurrence [23].

The strength of this study include the diary-based data which avoids memory recall problems, identification of key temperature oscillation related to headache and the removal of irrelevant, non-stationary trends by EMD methods, and the stratification of time series model by perception of temperature triggers. Most of prior investigations of weather factors associated with headache have been troubled by a lack of a longitudinal analysis [19], and may have substantial bias on assessing the exposure of patients to environmental risks. In the present analysis, patients and physicians were both blind to study protocols because the headache diary was made for clinical purposes, thus reducing the bias on both the patient and investigator sides.

Several limitations should be considered in interpreting these data. First, findings of this study were based on migraine patients visiting headache clinics; majority of patients were on migraine prophylactic agents and all on abortive treatments, which might have altered their response to temperature changes. However, current findings are consistent with our community-based study in which the study patients did not take prophylactic medications [23]. Second, the design of the study did not allow us to assess individual exposure to environmental risks. Third, this study only investigated temperature. Although weather as such also includes changes in barometric pressure, humidity, wind speed, or sunshine duration [20], the sub-tropical weather in Taiwan is dominant by temperature changes, which can be easily sensed by patients. Fourth, the study was based on patients with migraine; other types of headache sufferers may have different risk profiles seen in this study. Replication of these findings is required in other populations and geographical regions. In addition, because of the modest number of participants, this study only reported total headache incidence rather than the averaged intensity of headaches rating by patients. The perception of headache intensity may be more vulnerable to subjective bias and may therefore confound the current analysis.

## Conclusion

It has been a long debate about whether so many patients can be wrong about the association between weather and headache [19]? Using EMD approach to delineate the temporal relationship between temperature and headache, this study provides evidence to link the perception of temperature sensitivity and headache incidence in migraine patients. Those who reported temperature sensitivity in Taipei, Taiwan are more likely to have headache increase during the winter, particular for mild headaches.
